# Apical Hypertrophic Cardiomyopathy: A Fatal yet Underappreciated Variant of Hypertrophic Cardiomyopathy

**DOI:** 10.7759/cureus.51345

**Published:** 2023-12-30

**Authors:** Sherif Eltawansy, Lauren Klei, Steven Imburgio, Megan Decker, Ndausung Udongwo, Anas Alrefaee, Anton Mararenko, Nelson Lamarche

**Affiliations:** 1 Internal Medicine, Jersey Shore University Medical Center, Neptune Township, USA; 2 Cardiology, Jersey Shore University Medical Center, Neptune Township, USA

**Keywords:** implantable-cardioverter defibrillator, cardiac sudden death, ventricular tachycardia, syncope, hypertrophic cardiomyopathy

## Abstract

Hypertrophic cardiomyopathy (HCM) is a group of diseases affecting the left ventricle heart muscle that share a common feature of left ventricular hypertrophy without associated cardiac or systemic disorder. It was found to have a genetic basis with autosomal dominant mutations in the sarcomeric protein genes. Apical HCM is a rare subtype and underappreciated variant of HCM that primarily affects the apex of the heart. Apical HCM is dissimilar to classic HCM, with more challenges in diagnosis and inconsistent clinical course than other types.

We report a case of a 91-year-old female who presented with a syncopal episode. Workup revealed atypical nonclassic features. Her transthoracic echocardiogram revealed a "spade-like" configuration of the left ventricular cavity at end-diastole consistent with apical hypertrophic cardiomyopathy. The remaining of her workup was consistent with the apical hypertrophic cardiomyopathy as a reason for the syncopal episode on presentation.

Apical HCM is a distinct form of HCM that requires more attention among clinicians. In our case, the patient ended up having an implantable cardioverter defibrillator (ICD) for secondary prevention and a prescription of a beta blocker with a good outcome in her case.

## Introduction

Hypertrophic cardiomyopathy (HCM) is a common autosomal dominant disease of the myocardium characterized by significant variability in clinical and morphological presentations. It is distinguished by its localization to the left ventricle (LV) without involving other parts of the heart or any associated system disorder. There are five types of HCM, which can be enumerated as asymmetric septal, concentric, reverse septal, neutral, and apical. The asymmetric septal or basal is considered the classical type, while the apical is the rare type and the one we focus on in our case report [1,2]. The apical HCM is a rare and underappreciated variant of HCM that primarily affects the apex of the heart, also known as Yamaguchi syndrome which is a rare variant of HCM. It is characterized by abnormal thickening (hypertrophy) of the heart muscle, specifically affecting the apex of the LV [3].

Due to it primarily affecting the apex of the heart, there are diagnostic challenges that contribute to decreased detection. In addition, patients often present with nonspecific symptoms, further contributing to later diagnosis. Appropriate management of apical HCM is crucial, given that it is associated with an increased risk of atrial fibrillation, ventricular arrhythmias, and sudden cardiac death (SCD). Risk stratification for these complications is more challenging due to the phenotypic variation in apical HCM. Apical HCM is characterized by occurring at a later age, with a lack of genotype-phenotype correlation [4]. 

## Case presentation

A 91-year-old female with a medical history significant for left subclavian stenosis, chronic left bundle branch block, chronic obstructive pulmonary disease (COPD), and mild World Health Organization (WHO) group III pulmonary hypertension presented to the emergency department (ED) after an unwitnessed syncopal episode. She reported that she had been standing for approximately 20 minutes doing the laundry when she lost consciousness. She remembered waking up after an unknown downtime feeling dizzy and nauseous but denied any prodromal symptoms. She endorsed loss of consciousness and was unsure of the downtime when she was found by her family slumped over by the laundry machine. She stated that she had experienced four similar episodes in the six weeks that preceded this episode. She denied any identifiable triggers or prodromal symptoms. She reported living alone and was unsure of the downtime for each episode.

**Figure 1:**
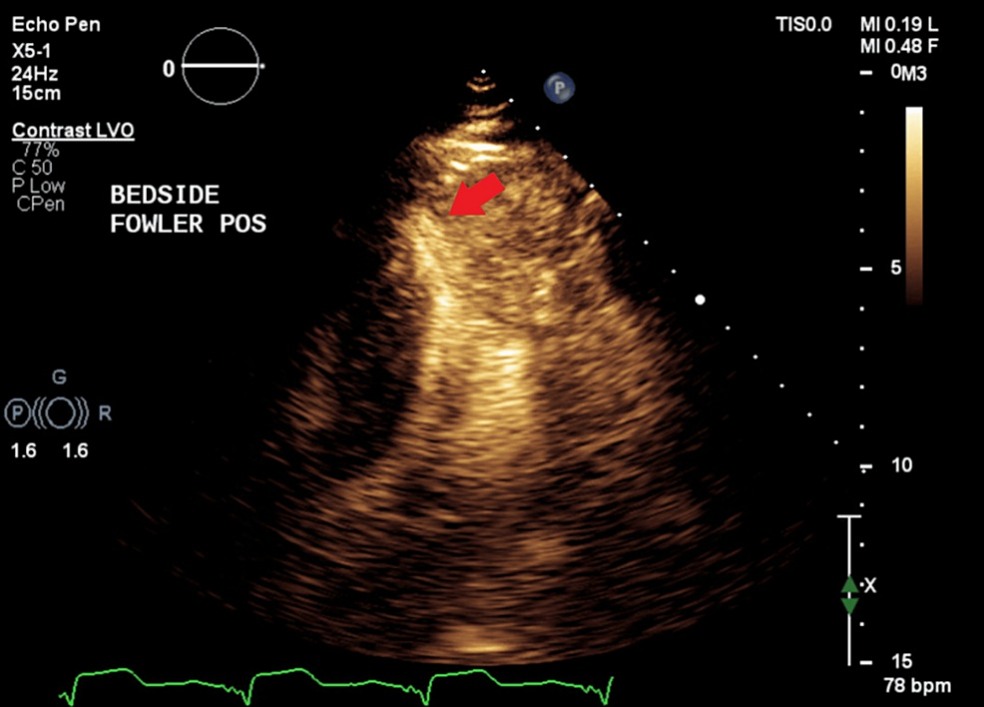
Transthoracic echocardiogram-apical view Apical thickening

On arrival at the ED, vital signs were measured with elevated blood pressure of 218/93 mmHg, with other vitals within the accepted range. The patient was on room air. The rest of the physical exam was unremarkable, with no signs of congestive heart failure like elevated jugular venous pressure. No murmur was heard. the electrocardiogram showed normal sinus rhythm with a known left bundle branch block. Telemetry revealed multiple episodes of non-sustained monomorphic ventricular tachycardia. High-sensitivity troponins were elevated and peaked at 652 ng/L (reference range: <34 ng/L). CT scan of the head showed no acute pathologies. Duplex ultrasound of the upper extremities showed no hemodynamically significant stenosis, and carotid ultrasound was also negative. The echocardiogram revealed an ejection fraction (EF) of 60-65% and a "spade-like" configuration of the LV cavity at end-diastole consistent with apical hypertrophic cardiomyopathy (Figure 1).

**Figure 2:**
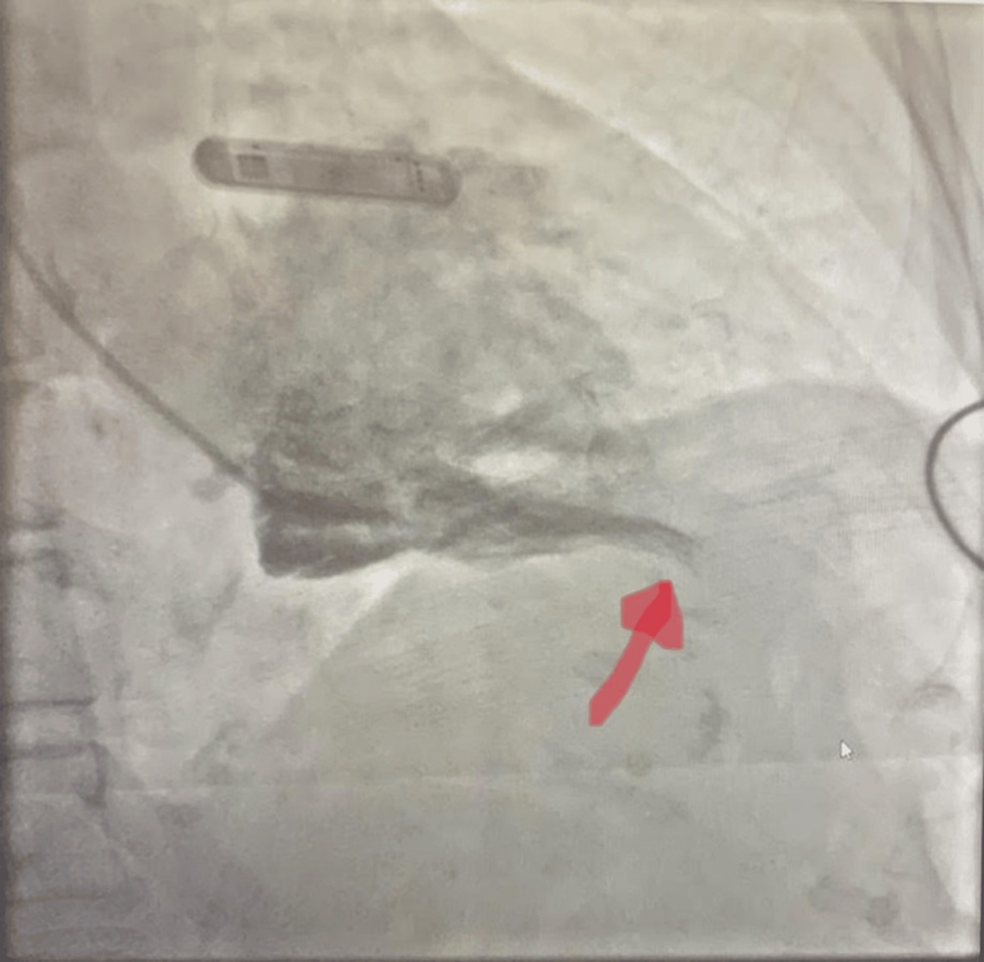
Left heart catheterization The left ventriculogram reveals overall normal cardiac size with what appears to be a hyperdynamic ventricle and failure of the apex to fill. This appears to be a possible apical hypertrophic cardiomyopathy. There was no gradient across the left ventricular outflow tract. Left ventricular end-diastolic pressure was normal.

Subsequent nuclear stress tests revealed mildly reduced EF and possible inferior/lateral ischemia vs. artifact. Given short run of wide complex tachycardia (non-sustained ventricular tachycardia) noted on telemetry during hospital stay and exertional dyspnea for six months with known coronary artery disease (CAD) based on a previous heart catheterization eight years ago, left heart catheterization was prompted. It was significant for mild non-obstructive coronary artery disease. Similar to the echocardiogram, the left ventriculogram demonstrated findings consistent with apical hypertrophic cardiomyopathy. Measurements of left ventricular pressures showed no evidence of outflow tract or intercavitary gradients (Figure 2).

In light of recurrent syncopal episodes and evidence of ventricular tachycardia, she received a dual-chamber defibrillator for secondary prevention and was started on metoprolol succinate 25 mg twice daily.

## Discussion

Apical HCM is a rare and potentially fatal variant of HCM that mandates a careful examination of cardiac imaging as well as a high degree of suspicion [5]. Unlike other morphologic variants of HCM, apical HCM does not cause left ventricular outlet obstruction (LVOT) but rather exists either with or without a mid-cavity obstruction or apical aneurysm. Therefore, patients with this cardiomyopathy are typically asymptomatic or present with mild symptoms. Patients presenting with nonspecific cardiac symptoms should be evaluated for apical HCM, especially in the presence of a positive family history. Some patients may present with more severe symptoms, such as syncope or sudden cardiac death due to ventricular tachycardia [6]. Management of apical HCM is dependent on the symptomatic presentation of patients and the natural history of the disease. Current pharmacological treatment approaches mirror those of classic HCM and are aimed at prolonging diastole. Beta-blockers or calcium channel blockers are most commonly used for mid-ventricular obstruction, cavity obliteration, and ventricular arrhythmias [7]. In patients whose symptoms are severe or refractory to medical treatment, apical myectomy may be considered. Finally, an implantable cardioverter defibrillator (ICD) is recommended for secondary prevention, but not primary prevention, in qualifying populations due to the low event rate. In our case, ICD placement was put to use in patient management, given the recurrent syncopal episodes and the non-sustained ventricular tachycardia episodes as seen on the telemetry monitor during the hospital stay. The syncope was not explained by other factors like left ventricular outlet obstruction. There was left ventricular thickening with a left ventricular mass of 298.35 g, as seen on the echocardiogram in our case. All these criteria warrant the use of ICD [8]. ICD placement has proven to reduce mortality in this population from 6% to 1% per year. Provider considers some risk factors before considering ICD placement, like age, report of syncope, ventricular arrhythmia, or left ventricular hypertrophy (LVH) ≥30 mm [9]. All these risk factors were found in our reported case. Further research and longitudinal data are necessary to develop appropriate risk stratification tools for SCD in apical HCM, investigate ICD usage, and guide overall management.

## Conclusions

Apical HCM is a rare and underappreciated variant of HCM that primarily affects the apex of the heart. Appropriate management of apical HCM is crucial given that it is associated with an increased risk of atrial fibrillation, ventricular arrhythmias, and SCD. Further research and longitudinal data are necessary to develop appropriate risk stratification tools for SCD in apical HCM, investigate ICD usage, and guide overall management. 
